# Stress cardiac magnetic resonance: follow-up of patients with intermediate-high cardiovascular risk

**DOI:** 10.1186/1532-429X-17-S1-P192

**Published:** 2015-02-03

**Authors:** Alberto Esteban-Fernández, Isabel Coma-Canella, Gorka Bastarrika-Aleman, Pedro M Azcárte-Aguero

**Affiliations:** Cardiology Department, Clínica Universidad de Navarra, Pamplona, Spain; Radiology Department, Clínica Universidad de Navarra, Pamplona, Spain

## Background

Stress cardiac magnetic resonance with adenosine (CMR-A) is a valid test to rule out myocardial ischaemia. We follow-up a cohort of patients with CMR-A due to suspected myocardial ischaemia.

## Methods

All the patients with a CMR-A were included between June 2009 and November 2012. The follow-up was done in outpatient cardiology clinic or by phone. We analyze the free-event survival considering as events: acute coronary síndrome (ACS), death for any cause, admission for heart failure (HF) or necessity of revascularization. The statistical analysis was made with SPSS 20.0.

## Results

239 patients were studied (180 male) with a mean age of 66±10 years old. One hundred and sixteen (48.5%) had previous coronary artery disease, with myocardial infarction in 68 patients. The reason for test referral were: several cardiovascular risk factors 52%, atypical chest pain 33%, typical chest pain 12%, and not conclusive previous test 3%.

**Figure 1:**
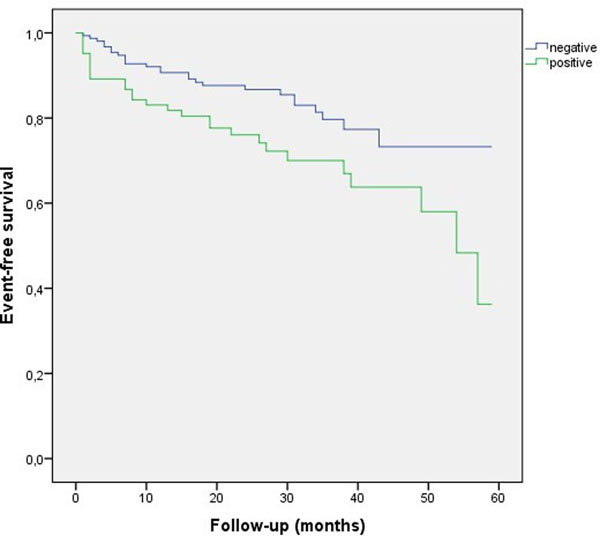
Figure 1

The CMR-A was positive for myocardial ischaemia in 83 patients (35%) and negative in 156 (65%). The follow-up median was 26 [0-59] months. 53 patients (22%) had events: 16 patients died (4 because of cardiovascular reasons), 26 had an ACS, 5 were admitted for HF and 21 needed invasive coronariography (18 PCI). There were statistical differences in the Kaplan-Meier survival curves (figure 1) between those with a positive result in the CMR-A test and those with a negative one (Long Rank test; p=0.021).

## Conclusions

In this cohort of patients with an intermediate-high cardiovascular risk, with CMR-A, those with a negative result have fewer events in the follow-up.

## Funding

There is not any specific funding to support this trial.

